# Diabetes and TelecommunicationS (DATES) study to support self-management for people with type 2 diabetes: a randomized controlled trial

**DOI:** 10.1186/s12889-018-6136-8

**Published:** 2018-11-12

**Authors:** Ebaa Al-Ozairi, Katie Ridge, Etab Taghadom, Nicole de Zoysa, Clare Tucker, Kurtis Stewart, Daniel Stahl, Khalida Ismail

**Affiliations:** 1 Faculty of Medicine, Kuwait University, P. O. Box 24923, 13110 Safat, PO Kuwait; 2 Institute of Psychiatry, Psychology and Neurosciences, King’s College London Weston Education Centre, 10 Cutcombe Road, London, SE5 9RJ UK; 3 Dasman Diabetes Institute, Al Kuwayt, Kuwait

**Keywords:** Type 2 diabetes, Randomised controlled trial, Telehealth, Text messages, Motivational interviewing, Psychological intervention

## Abstract

**Background:**

The increasing prevalence of type 2 diabetes and suboptimal glycaemic control in Kuwait requires novel, wide-reaching, low-cost interventions to motivate and mobilise individuals towards more effective self-management. More than 2 million people in Kuwait own mobile phones. We will test whether automated personalised health text messages based on principles of motivational interviewing and are responsive to biodata delivered remotely is potentially effective in improving glycaemic control compared to usual care.

**Methods:**

This is a two-arm parallel single-blind randomised controlled trial of 572 individuals with type 2 diabetes in Kuwait. We will develop a culturally appropriate database of text messages supporting positive lifestyle changes in type 2 diabetes. A computer programme will deliver over 400 text messages over a 12-month period using algorithms which provide participants with information on diet and physical activity as well as personalised messages regarding motivators to change behaviours. Individuals aged 18–75 years with established type 2 diabetes who are fluent in Arabic or English and officially resident in Kuwait will be identified via screening of hospital diabetes clinic and primary care practices and invited to participate. A sample of 572 participants will be randomised to usual care or usual care plus the DATES text message intervention. Randomisation will be conducted by an independent Clinical Trials Unit and researchers collecting baseline and outcome data will be blinded to treatment allocation. The primary outcome is change in HbA1c and weight at 12 months in both study arms. Secondary outcomes will include changes in physical activity, fasting lipids and quality of life in both study arms.

**Discussion:**

The potential of mobile phones in improving diabetes self-care in settings with a high prevalence of diabetes and widespread mobile phone usage has face validity. Mobile phones and text messaging are an understudied virtual communication media which can deliver discrete focused psychological support to motivate and enable diabetes self-care changes.

**Trial registration:**

ISRCTN10342151. 11/03/2015.

**Electronic supplementary material:**

The online version of this article (10.1186/s12889-018-6136-8) contains supplementary material, which is available to authorized users.

## Background

Diabetes mellitus is one of the most common non-communicable diseases with an estimated global prevalence of 9% in 2014 [1–3]. The age standardized prevalence of diabetes in individuals over the age of 18 years in Kuwait is 20.1%; the second highest prevalence of type 2 diabetes reported in all high-income countries by the World Health Organisation [3]. The increased prevalence of type 2 diabetes in countries such as Kuwait has been attributed in part to significant socioeconomic change, which can enable a more sedentary lifestyle and increased intake of high calorie food [4].

Complications of diabetes are well understood. Persistent hyperglycaemia can cause macrovascular complications such as cardiovascular and cerebrovascular disease and micro-vascular complications including nephropathy, neuropathy and retinopathy. Type 2 diabetes is managed by achieving optimal glycaemic control [1] where life-long self-care tasks including physical activity, a healthy diet, weight management, and self-monitoring of blood glucose is combined with the administration of oral medication where necessary and increasingly insulin therapies [1]. Structured education [5], lifestyle modification [6] and intensive medical regimens [7, 8] have been shown in research settings to be efficacious in improving glycaemic control. Many people with type 2 diabetes struggle to adhere to self-care tasks [9] and to attend medical appointments. Patients who miss more than 30% of their scheduled appointments within 1 year tend to have HbA1c levels that are 0.7% higher than those who keep all appointments [10]. Younger individuals are more likely to have sub-optimal glycaemic control as do those who are more obese [11]. In Kuwait, there is a severe shortage of diabetes educators, dieticians and clinical psychologist. Diabetes accrues the highest health care costs in Kuwait.

The prevalence of depression is increased 2-fold in people with diabetes compared to the general population [12]. Depression in diabetes is associated with reduced self-care [13, 14], poorer glycaemic control [15], and increased mortality [16]. Individuals with suboptimal glycaemic control can also experience psychological difficulties such as problems accepting and adjusting to a diagnosis, fears about diabetes complications, anxieties surrounding blood sugar testing and injecting, fears of hypoglycaemia, and concerns about body image [17]. In recent years, a rising incidence of anxiety and depression has been noted in Arab states, particularly in individuals with chronic illness [18].

Psychological treatments are talking therapies where the patient and the therapist use the therapeutic alliance or collaboration between them to bring about change in thoughts, emotions and behaviours. A systematic review of psychological treatments in diabetes found a pooled estimate of 0.8% improvement in HbA1c [19]. However, the review found rigorous studies to be lacking and most interventions used face to face interaction with a clinical psychologist. In some settings around the world, including Kuwait, there is a dearth of clinical psychologists, especially those with expertise in health psychology and chronic medical conditions.

Motivational interviewing (MI) is a patient-centered counselling approach designed to support people during behavior change [20]. It aims to strengthen an individual’s motivation and movement towards a specific goal by eliciting and exploring his/her own arguments for change and is characterized by collaboration (as opposed to confrontation); evocation (as opposed to didactic reasoning) and patient autonomy (as opposed to authoritative style). Systematic reviews and meta-analyses have consistently shown that MI techniques have a moderate effect on diet and exercise (effect sizes (d) of 0.53 standard deviations in four RCTs) [21]. In another meta-analysis, there was a large pooled effect observed for weight reduction (d = 0.72) with a smaller but still significant pooled effect in cholesterol reduction (d = 0.27) although the number of trials were few [22].

Conventional psychological treatments are delivered face-to-face, have a set number and duration of sessions, require significant resources in personnel and patient commitment to attend. They are difficult to incorporate into routine clinical practice and patient uptake can be poor [23]. With the increasing burden of type 2 diabetes, there is a growing need to find novel approaches to delivering psychological support which motivates patients in their diabetes self-care more effectively [24]. E-technologies offer an opportunity to deliver psychological care in a virtual setting.

E-health is the use of electronic information and communication technology, particularly the internet, to improve or enable health and health care [25]. Benefits of e-health include the rapid delivery of health information without attendance at a medical appointment, increased anonymity and acknowledgement of the stigma associated with diabetes and accessing sensitive health information [26]. M-health refers to the use of mobile devices such as phones, for health processes. Motivational messages and behaviour-change methods used in face-to-face support can be modified for delivery via mobile phones with the content tailored to the participant and can be interactive where the participant can seek help when required [27].

Text messages aimed at improving glycaemic control have been used successfully in individuals with impaired glucose tolerance with messages tailored to the individual’s perceived motivation to change their behaviour [28]. In individuals with established type 2 diabetes, a cluster randomised controlled trial of 163 patients who received educational and motivational messages regarding diabetes self-care reported a significant decrease in HbA1c when compared with usual care [29]. However, the intensity and content of these messages is not described neither is the underlying psychological theory informing their content. A meta-analysis of 22 studies found that mobile phone interventions for diabetes self-management reduced HbA1c values by a mean of 0.5% [30]. However, this study focused on type 1 and type 2 diabetes and intervention format was varied. There have been no RCTs testing the effectiveness of text messages matched to biodata such as physical activity levels, dietary records and thoughts and feelings supported by wearable technologies in improving glycaemic control in type 2 diabetes.

Motivational messages delivered by m-health have been shown to be an effective adjunct to interventions supporting smoking cessation, dieting and increased physical activity and exercise, especially with the use of pedometers. In Kuwait, more than 2 million people depend on mobile technology. Kuwaitis have been estimated to use their mobile phone for more than 100 min per day on average.

### Aims and objectives

The primary objective of this study is to compare the effectiveness of an automated motivational text message intervention delivered by mobile technology with usual care in improving glycaemic control in type 2 diabetes. Secondary objectives include the examination of psychological, sociodemographic and biological predictors of improved glycaemic control, and to develop a manual that can be adapted and translated to other clinical settings.

## Methods

**Fig. 1:**
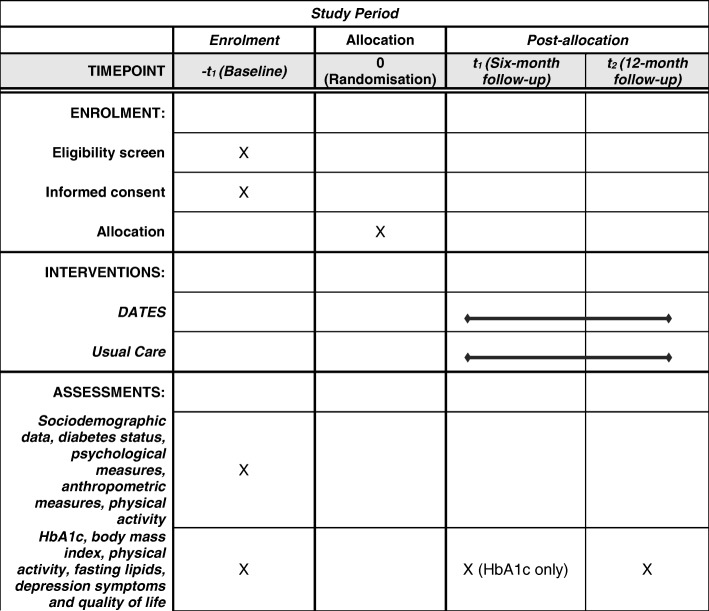
SPIRIT schedule of enrolment, interventions, and assessments for the DATES trial

This is a single centre two-arm parallel randomized controlled trial comparing usual care with mobile motivational care. Eligible participants will be adults with poorly controlled type 2 diabetes who are registered at the Dasman Diabetes Institute, Kuwait and affiliated primary care centres, as well as from non-affiliated primary care centres that have electronic diabetes registers who agree to participate. Type 2 diabetes will be defined according to current World Health Organisation criteria. Poorly controlled diabetes will be defined as having at least one HbA1c value of > 8% in the preceding 12 months and also at recruitment despite standard care defined as the offer of at least 2 diabetes clinic reviews in the same time period. Figure 1 shows the SPIRIT flow diagram for the study.

### Inclusion criteria

i). On diet and/or oral antidiabetic agents and/or insulin, all on stable dose for the past 3 months and not titrating their doses.
ii). Age 18–75 years.
iii). Fluent in spoken Arabic or English with a minimum reading age in Arabic or English of 7 years.
iv). Officially resident in Kuwait.
v). In possession of and uses a mobile phone.

### Exclusion criteria

i). Duration of type 2 diabetes less than 1 year.
ii). Pregnant women or women planning pregnancy.
iii). Individuals with severe mental illnesses such as psychosis, learning difficulties, dementia excluded from the medical records and by checklist from the physician.
iv). Individuals with advanced cancer or diabetes complications (renal failure as measured by eGFR < 50, above ankle amputation, registered partially blind) or terminal conditions.
v). Inability or unwillingness of individual or legal guardian/representative to give written informed consent.
vi). Individuals with another study participant living in the same home.

A research worker will screen the Dasman diabetes register and participating primary care clinics to identify all eligible individuals. Potential participants will be invited to participate by invitation in the post or telephone call. If the patient is interested, the study research worker will invite the patient for screening. Eligible persons will be given written information sheets and a verbal explanation of the study, after which written informed consent will be requested. The number of patients who do not meet the study criteria or do not give informed consent or for whom information is incomplete will be recorded for the purposes of assessing participation bias.

Baseline data collection will generally follow those of Bayley et al. [31] and include:

i). Sociodemographic information: age, gender, nationality, native language, educational attainment, occupation, marital status and years in Kuwait.
ii). Diabetes status: duration of type 2 diabetes, current HbA1c, fasting lipid status, blood pressure, body mass index, macrovascular and microvascular complication status and medication history.
iii). Psychological measures: Hospital Anxiety and Depression Scale (HADS) [32], Patient Health Questionnaires 9 (PHQ-9) [33], and the Problem Areas in Diabetes measure of diabetes-specific quality of life (PAID) [34]. Motivation to change behaviours will be assessed using a readiness ruler which is a visual analogue scale from 1 to 5 asking patients to score a) their confidence in their ability to change and b) their willingness to change.
iv). Anthropometric measures, as in Bayley et al. [31]: Weight will be measured in light clothing, without shoes, on calibrated digital weighing scales to 0.01 kg for weight. Height will be measured to 0.1 cm using SECA stadiometers with the supported stretch stature method. Waist circumference will be measured horizontally halfway between the lowest rib and the upper prominence of the pelvis using a non-extensible steel tape against the bare abdomen. Blood pressure and resting heart rate will be measured with the Omron 1025 digital BP monitors using standardised procedures.
v). Physical activity will be measured using a pedometer (number of steps per day) and the International Physical Activity Questionnaire (IPAQ) [35].

Randomisation of participants will be conducted by the data manager from an independent Clinical Trials Unit using computer generated randomisation blocks of random sizes. Allocation concealment will be ensured, as the randomisation list will be held in a password-locked computer and ACCESS programme. The data manager can only reveal to himself and then the researcher the next allocation after entering the details of the next participant recruited. As this is a complex intervention, it is not possible to conceal to the allocation to the participants, but outcome assessors and technicians will be blind to the allocation for the primary and secondary outcomes. There is a small inevitable risk that allocation will be revealed to the outcome assessors which we will aim to minimise by asking participants not to reveal their allocation.

### Study interventions

#### Usual care

All participants will receive usual diabetes care from their usual diabetes clinical service. This includes face-to-face clinical appointments with the diabetologist, diabetes specialist nurse and dietician, prescribing of medication, lifestyle advice and counselling using adult learning education techniques. In addition to control for the attention of receiving more text messages, the usual care group will also receive weekly text messages from the study group such as messages thanking them for participation or study rationale. Both groups will also receive the wearable technology with standard manufacturer instructions for their use. Both groups will also have access to a telephone hotline run by the research nurse to provide additional clinical support should any issues arise as a result of text messages received.

#### Usual care plus DATES intervention

In addition to usual care as described above, participants will receive motivational text messages to support them in making diet and exercise changes. A proportion of these messages will be based on biodata sent from wearable technology (a wrist-band pedometer collecting data on physical activity). The theoretical framework underlying the text messages is based on motivational interviewing [20] and principles drawn from cognitive behaviour therapy [36, 37].

The messages will contain both standardized, personalized and responsive messages. Standardised text messages will be delivered at set times. Responsive text messages will be tailored to participant requests (e.g. help, crave) and to the biodata received.

The key features of the DATES package will be:

i) A personalized intervention: At baseline assessment, participants will be asked to identify their top three reasons for wanting better diabetes control. Participants will be provided with prompts if they are unable to generate their own reasons. Prompts might include: to stay healthy for family, to avoid complications, to live longer, to keep one’s job or to feel physically better. These personal goals will be used to develop approximately 100 text messages which relate to an individual’s motivation to change.
ii) Intensity: Participants will receive 4 text messages a day for 12 months: two standard text messages that everyone receives; one personalized message based on the nature of the personal motivator for improved diabetes control; and 1 messages in response to the biodata received.
iii) Targeted behaviours: this will be a rolling programme of automated messages delivered to all participants in the intervention group as follows:
  Months 1–3: Diet, weight and healthy eating
  Months 4–6: Physical activity and exercise
  Months 7–12: Diet, weight, healthy eating, physical activity and exercise
iv) Responsiveness: Participants will be invited to text HELP, CRAVE or LAPSE when they are feeling particularly vulnerable to a relapse. CRAVE will indicate that the participant is thinking about pursuing an unhealthy behaviour (e.g. eating a high calorie food or avoiding their exercise regime) but have not acted upon it yet. LAPSE will indicate that patients have acted upon their cravings and need support to reengage with their good intentions. (See Table 1 for examples of these messages).
v) Biofeedback: data received from the wearable technology on activity levels will be coded by a computer software into optimal (eg 10 k steps a day), suboptimal (eg 3 k steps/day) and low levels (< 3 k steps/day) and text messages designed to reinforce or encourage increase in activity sent in response (see Table 1 for examples).
vi) Availability of information: Participants can call the study Helpline for immediate advice between 9 am and 12 am should they have any medical queries. Participants will be provided detailed information including the Helpline phone number, the text codes and definitions (i.e. HELP, CRAVE and LAPSE).

**Table 1:** Sample messages

STANDARDISED MESSAGES	
‘Trying a new activity can give you a sense of achievement’	
‘See what small and realistic changes you can make to your diet today.’	
PERSONALISED MESSAGES	
‘Taking care of your health is setting a fantastic example to your children’ (if family is motivator)	
‘Better skin, a glowing complexion and better shape are within reach when you look after your diabetes’ (if appearance is motivator)	
RESPONSIVE MESSAGES	
‘Think back to all the good health decisions you’ve made over the last few weeks; really hold on to that feeling of achievement and wellbeing and step away from this temptation.’	
‘Keep calm, know that lapses are normal and find the strength within you to set yourself a new, realistic goal for the week ahead’.	
BIOFEEDBACK MESSAGES	
It looks like you are making an effort to move around and perhaps you are wondering how else you can fit in a few more steps.... it only takes 10 min to walk 1000 steps and you have already improved your step count massively! (for < 3 k steps)	

### Study management

Standard Operating Procedures will provide detailed instructions for training, collecting data to high methodological standard and maintaining study records. We will monitor adverse side effects of the DATES intervention. As this is a non-pharmacological intervention adverse effects are expected to be few. The literature suggests a small risk of increased falls, thumb strain and accidents if using a mobile phone excessively but in a recent RCT of m-health for smoking cessation, there was no reported increase in adverse events in the intervention group. We will ask about these in the preceding month at baseline and at the end of the 12-month study. We will record if the patient’s medication has been changed, becomes pregnant or has a fatal or non-fatal event during the course of the intervention. We will be able to monitor the use of DATES by markers such as frequency of responsive texts and use of the clinical hotline.

The primary outcome will be HbA1c at baseline and 12 months in both study arms. The secondary outcomes will include body mass index, physical activity, fasting lipids, depression questionnaires and quality of life at baseline and 12 months in both study arms. We will measure HbA1c at 6 months to examine the rate of change in glycaemic control and to minimise attrition.

### Statistical methods

Baseline characteristics will be presented using means and their standard deviations (SD). Where consent is given, baseline characteristics of individuals who decline participation or withdraw from the study will be collected and compared with that of participants. An intention to treat analysis will be conducted using STATA 13. The differences in treatment effect between the two arms at 12 months will be analysed using mixed effects models with pre-randomisation values as a covariate [38] This approach provides valid inferences under the assumption that the missing data mechanism can be ignored (or missing at random). Sensitivity analyses using baseline variables which predict missingness at follow-up in the analyses model will be performed. We will also conduct an analysis of moderators of treatment effectiveness.

We assumed a conservative mean difference of 0.5% in the DATES group compared with usual care. We also estimated that the standard deviation of the mean difference in each group was 1.65 based on our systematic review [19]. At a power of 90%, type 1 error rate of 0.05 (two-tailed), randomisation ratio of 1:1 we estimated using the sampsi function in STATA 10 that we will need 229 participants in each arm. Assuming a 20% dropout rate, the total sample required is n = 572. See the Additional file 1 for the detailed statistical plan.

### Data collection and quality assurance

Data files will be analysed in the data centre for final checking. All data will be entered twice and the project manager will ‘clean’ the database before analysis (i.e. checking for discrepancies and outliers). Data will be checked every six months with results relayed to a Trial Steering Committee and Data Monitoring and Ethics Committee for trial supervision. Report of any violations to the trial protocol will be reported.

## Discussion

The potential of m-health in diabetes self-care in settings with a high prevalence of diabetes and widespread mobile phone usage has face validity. M-health is an understudied virtual communication media which can deliver discrete focused psychological support to motivate and enable diabetes self-care changes. It is cheap, adaptable to different cultural norms and has a wide coverage. We aim to test whether adjunct motivational interviewing delivered by m-health improves glycaemic control in a Kuwaiti diabetes population with suboptimal glycaemic control compared to usual care avoiding the use of biofeedback system or complicated software, so it can be reproduced in countries with limited resources.

## Additional file

Additional file 1: DATES analysis plan 2018-07-11. Analysis plan of “Diabetes and Text Messaging Study to support self-management for people with type 2 diabetes: a randomized controlled trial” (The DATES trial). Description of data: This is the full analysis plan, detailing all planned statistical analyses for the DATES trial, to accompany the protocol. (DOCX 126 kb)

## Abbreviations

E-health: Electronic health
HbA1c: Haemoglobin A1c/Glycosylated haemoglobin
M-health: Mobile health
