# Supplementary material for: Diabetes and TelecommunicationS (DATES) study to support self-management for people with type 2 diabetes: a randomized controlled trial
Source: BMC Public Health. 2018 Nov 12;18:1249. doi: 10.1186/s12889-018-6136-8 (PMC6233607; doi:10.1186/s12889-018-6136-8)
Supplement: Supplementary file 1 — DATES analysis plan 2018-07-11. Analysis plan of “Diabetes and Text Messaging Study to support self-management for people with type 2 diabetes: a randomized controlled trial” (The DATES trial). Description of data: This is the full analysis plan, detailing all planned statistical analyses for the DATES trial, to accompany the protocol. (DOCX 126 kb) [file 12889_2018_6136_MOESM1_ESM.docx]

SUPPLEMENTARY MATERIAL

Analysis plan

of

“Diabetes and Text Messaging Study to support self-management for people with type 2 diabetes: a randomized controlled trial” (The DATES trial)

Table of Contents

[1. Brief description of the trial 4](#_Toc519064410)

[1.1. Research Objectives 4](#_Toc519064411)

[1.2. What is the trial design? 4](#_Toc519064412)

[1.3. Randomisation and allocation concealment 5](#_Toc519064413)

[1.4. Setting 5](#_Toc519064414)

[1.5. Target population 5](#_Toc519064415)

[1.6. Eligibility screening 6](#_Toc519064416)

[Patients from same household 7](#_Toc519064417)

[1.7. Baseline measures 7](#_Toc519064418)

[Sociodemographic 7](#_Toc519064419)

[Diabetes Status 7](#_Toc519064420)

[Psychological functioning 7](#_Toc519064421)

[Anthropometric measures 8](#_Toc519064422)

[Physical activity 8](#_Toc519064423)

[1.8. Follow-up measures 8](#_Toc519064424)

[1.9. Duration of treatment period 9](#_Toc519064425)

[1.10. Sample size and power calculations 9](#_Toc519064426)

[1.11. The original proposed statistical analyses of main outcome variables 9](#_Toc519064427)

[2. Data Analysis Plan: description 10](#_Toc519064428)

[2.1. Description of available data 10](#_Toc519064429)

[2.2. Recruitment: GP and Participant Flow 10](#_Toc519064430)

[2.3. Baseline comparability of randomised groups 12](#_Toc519064431)

[2.4. Adherence to the protocol 12](#_Toc519064432)

[2.5. Loss to follow-up and other missing data 13](#_Toc519064433)

[Missingness pattern of main outcome variable 13](#_Toc519064434)

[2.6. Adverse event reporting 13](#_Toc519064435)

[3. Statistical Data Analyses Plan: Comparison of HBA1c between intervention groups (Primary outcome analysis) 14](#_Toc519064436)

[3.1. General comments 14](#_Toc519064437)

[3.2. Comments to Study design 14](#_Toc519064438)

[3.3. Overview of analyses of primary outcome: 15](#_Toc519064439)

[Primary Intention to treat analysis 15](#_Toc519064440)

[Sensitivity of results due to potential imbalance of confounders at baseline 17](#_Toc519064441)

[Sensitivity of results to missing data 17](#_Toc519064442)

[Sensitivity of results due to insulin uptake during the treatment period 19](#_Toc519064443)

[Complier average causal effects (CACE) 20](#_Toc519064444)

[Subgroup analyses 20](#_Toc519064445)

[4. Secondary outcome analyses 21](#_Toc519064446)

[5. Considering incomplete measures of scale (questionnaires) data 21](#_Toc519064447)

[6. Adverse Events 22](#_Toc519064448)

[7. Compliance TO PROTOCOL 23](#_Toc519064449)

[8. References 24](#_Toc519064450)

#

# Brief description of the trial

## Research Objectives

**Main objectives**

The overall aim is to compare in a randomized controlled trial (RCT) the effectiveness of motivational interviewing for improving diabetes self-management adapted into text messages delivered by mobile phones and internet technology (M-health; DATES intervention) in improving glycaemic control in people with type 2 diabetes and suboptimal glycaemic control compared to usual care over a 12-month follow-up period.

***Primary objective:***

To compare in a RCT the effectiveness of the DATES intervention to usual diabetes care in improving glycaemic control (measured as HbA1c) in people with poorly controlled type 2 diabetes over a 12-month period.

***Secondary objectives:***

i) The secondary outcomes are body mass index (BMI), physical activity levels (number of steps per day and self-reported physical activity using the International Physical Activity Questionnaire [IPAQ]), fasting lipids, depressive symptoms using the Patient Health Questionnaire (PHQ-9), and diabetes-specific quality of life using the Problem Areas in in Diabetes measure (PAID). We will examine whether the DATES intervention is associated with changes in these outcomes over the 12-month study period compared to usual care.

ii) To examine whether baseline psychological (depression), sociodemographic (gender) or biological (duration of diabetes, insulin use) factors moderate the improvement in glycaemic control.

## What is the trial design?

This is a two-arm parallel multi-centre RCT comparing usual care with mobile and motivational care (m-health) to usual care alone.

The two arms consist of:

1. **Usual care:** Standard diabetes care.

2. **Usual care plus DATES Intervention:** Motivational interviewing for improving diabetes self-management adapted into text messages delivered by mobile phones and internet technology (m-health) in addition to standard care.

Primary and secondary outcome data will be collected at baseline before randomisation and at six (except for some secondary outcomes, see Table 1) and 12 months post-randomisation.

## Randomisation and allocation concealment

Randomisation of participants will be conducted by the data manager from an independent Clinical Trials Unit using a computer-generated randomisation blocks of random sizes. Allocation concealment will be ensured as the randomisation list will be held in password-locked computer and ACCESS programme. The data manager can only reveal to himself and then the researcher the next allocation after entering the details of the next participant recruited. As this is a complex intervention, it is not possible to conceal to the allocation to the participants, but outcome assessors and technicians will be blind to the allocation for the primary and secondary outcomes. There is a small inevitable risk that allocation will be revealed to the outcome assessors which we will aim to minimise by asking participants not to reveal their allocation.

## Setting

The setting will be the Dasman Diabetes Institute (DDI) which is the only tertiary centre for diabetes care in Kuwait, its four affiliated primary care clinics and those diabetes primary care clinics in Kuwait that have electronic diabetes-specific databases and agree to participate in the study.

## Target population

Adults with poorly controlled type 2 diabetes mellitus who are registered at DDI or the primary care centres affiliated with DDI. Type 2 diabetes will be defined according to current World Health Organisation criteria. Poorly controlled diabetes will be defined as having at least one HbA1c value of > 8% in the preceding 12 months and at recruitment despite standard care defined as the offer of at least two diabetes clinic reviews in the same time period.

## Eligibility screening

**Inclusion Criteria**

i) On diet and/or oral anti-diabetes agents and/or insulin on a stable dose for the past 3 months and not titrating their dosages.

ii) Age 18-75 years to increase the representativeness of the sample and to be inclusive of all ages.

iii) Fluent in spoken Arabic or English and have a reading age in Arabic or English of a minimum age 7 years.

iv) Officially resident in Kuwait.

**Exclusion Criteria**

i) Duration of type 2 diabetes less than one year as these patients are still coming to terms and adjusting to the diagnosis and the self-care roles.

ii) Women who are gestational or planning pregnancy as they have specialist diabetes care needs

iii) People with severe mental illnesses such as psychosis, learning difficulties, dementia excluded from the medical records and by checklist from the physician.

iv) People with advanced cancer or diabetes complications (renal failure as measured by eGFR <50, above ankle amputation, registered partially blind) or terminal conditions.

v) Inability or unwillingness of individual or legal guardian/representative to give written informed consent.

vi) Multiple insulin doses regimens as this will increase the need for more clinical resources to provide a more intensive biofeedback service requiring more clinician support. The purpose of the DATES study is to test whether a stand-alone psychological intervention delivered in a virtual media with minimum clinical support can be effective as an adjunct to usual diabetes care.

vii) People who do not have access to a mobile phone or the Internet.

### Patients from same household

To avoid a contamination of treatment of persons who are living in the same household only one person per household (defined as family and co-resident first and second-degree relatives) will be randomized to a treatment. If two or more persons from one household is recruited at the same time only one person will be randomly selected. The patient ID numbers will be sent to an independent researcher who will use a random number generator to select one person for randomization and inform the researcher. If a patient of a household was randomized to a treatment, then no other household members will be considered for recruitment for the duration of the study.

## Baseline measures

These will be collected prior to randomisation.

### Sociodemographic

Data age, gender, nationality, native Arab speaking, educational attainment, occupation, marital status, years in Kuwait

### Diabetes Status

Diabetes status at baseline: duration of type 2 diabetes (years), HbA1c, fasting lipid status, blood pressure, body mass index, macrovascular and microvascular complication status, medication history.

### Psychological functioning

Hospital Anxiety and Depression Scale (HADS), Patient Health Questionnaires 9 (PHQ-9), and the Problem Areas in Diabetes measure of diabetes-specific QoL (PAID). All are validated and have been translated by the PI. Motivational status is measured by readiness ruler which is a visual analogue scale with five categories from 1-5 that asks patients to score their a) their confidence in their ability to change and b) their willingness to change.

### Anthropometric measures

Weight, body fat, height, BMI, waist circumference, blood pressure, and resting heart rate.

### Physical activity

Physical activity will be measured objectively using a pedometer (number of steps per day) which was worn for one week before randomisation. We will ask participants to keep a log of activities including sedentary ones to assist with the qualitative interpretation of the data.

## Follow-up measures

Updated sociodemographic, biomedical, lifestyle and psychological data will be collected at 6 and 12 months follow-up after baseline by an independent researcher who is blind to the treatment condition

| Table 1. Data collection timeline. | | | |
| --- | --- | --- | --- |
|  | Baseline (pre-randomisation) | Six-month follow-up | 12-month follow-up |
| HBA1c | X | X | X |
| Insulin use/status | X | X | X |
| Physical activity (number of steps and self-report [IPAQ]) | X | X | X |
| Depressive symptoms (PHQ-9) | X |  | X |
| Diabetes-specific QoL (PAID) | X |  | X |
| Motivational status | X |  | X |
| Weight | X | X | X |
| Height | X |  |  |
| BMI | X | X | X |
| Waist circumference | X |  | X |
| Blood pressure | X |  | X |
| Resting heart rate | X |  |  |
| Fasting lipid status, | X |  | X |
| Macrovascular complication status | X |  |  |
| Microvascular complication status | X |  |  |
| Medication history | X |  | X |

## Duration of treatment period

The duration of the treatment is 12 months.

## Sample size and power calculations

We expect a conservative mean difference of 0.5% in the DATES group compared with usual care after 12 months. We also estimated that the standard deviation of the mean difference in each group was 1.65 based on our systematic review. A difference of 0.5 after 12 months would therefore corresponds to a small effect size of d=0.3. At a power of 90%, type 1 error rate of 0.05 (two-tailed), randomisation ratio of 1:1 we estimated using the sampsi function in STATA 10 that we will need 229 participants in each arm. Assuming a 20% dropout, the total sample required is n=572.

## The original proposed statistical analyses of main outcome variables

A description of the sample will be presented using means and their standard deviations (SD). Baseline characteristics of refusers and drop-outs will be compared with participants who complete the study. An intention to treat analysis will be conducted using STATA 15. The differences in treatment effect between the two arms at 12 months will be analysed using mixed effects models with pre-randomisation values as a covariate. This approach provides valid inferences under the assumption that the missing data mechanism can be ignored (or missing at random). Subgroup analyses will be conducted to assess if any baseline characteristics modified the outcome.

# Data Analysis Plan: description

The analysis will follow the guidelines of the CONSORT statement for RCTs. The trial statistician will remain blind whenever possible until the main analyses are complete.

## Description of available data

The patterns of availability of baseline and 6 and 12 months follow-up data will be summarised overall and separately for the two intervention groups for each assessment visit and scale; presented by descriptive summary statistics of clinical and demographical assessments.

QQ plots and histograms will be used to assess data distribution of continuous measures. Appropriate summary statistics will be applied, mean and standard deviation for all normally distributed measures; median and quartiles for skewed distributions and ordinal data. Categorical outcomes will be described using both the number and proportion (percentage).

## Recruitment: GP and Participant Flow

Flow of main recruitment type (DDI/GP seen) *and* individual participants through each stage will be summarized in a CONSORT diagram, including for each group report the numbers of participants randomly assigned, receiving intended treatment, completing the study protocol, and analysed for the primary outcome

We will describe protocol deviations from the study as planned together with reasons. Participant throughput will be summarised in a CONSORT diagram, including the stages of enrolment, allocation, follow-up and analysis.

**Figure 1. CONSORT 2010 Flow Diagram**

Follow-Up

Analysed (n= )
♦ Excluded from analysis (give reasons) (n= )

Analysis

Analysed (n= )
♦ Excluded from analysis (give reasons) (n= )

Lost to follow-up (give reasons) (n= )

Discontinued intervention (give reasons) (n= )

Lost to follow-up (give reasons) (n= )

Discontinued intervention (give reasons) (n= )

Enrolment

Allocated to intervention (n= )

♦ Received allocated intervention (n= )

♦ Did not receive allocated intervention (give reasons) (n= )

Allocated to intervention (n= )

♦ Received allocated intervention (n= )

♦ Did not receive allocated intervention (give reasons) (n= )

Randomized (n= )

Excluded (n= )

♦  Not meeting inclusion criteria (n= )

♦  Declined to participate (n= )

♦  Other reasons (n= )

Assessed for eligibility (n= )

Allocation

From: <http://www.consort-statement.org/consort-statement/flow-diagram0/>

Schulz KF, Altman DG, Moher D, for the CONSORT Group. CONSORT 2010 Statement: updated guidelines for reporting parallel group randomised trials. BMC Medicine 2010, 8:18. (24 March 2010)

## Baseline comparability of randomised groups

Participant-level baseline variables will be described both overall and by randomised group. No statistical significance tests or confidence intervals will be calculated for the difference between randomised groups on any participant level baseline variables to assess for baseline differences. The randomisation of intervention groups to participants should have ensured that any imbalance over all measured and unmeasured baseline characteristics is due to chance. In a secondary sensitivity analyses we will include potential confounding variables which differ substantially between the two arms. Variables measured at baseline which may affect primary outcome will be assessed for potential imbalance. The following variables will be assessed:

1. Age
2. Gender
3. Kuwait/non-Kuwait citizen
4. Education status
5. Marital status (Married/Divorced/Alone/Widowed)
6. Smoking status
7. Duration of Diabetes
8. BMI
9. Insulin use
10. HbA1c
11. PHQ9
12. PAID
13. IPAQ
14. HADS

## Adherence to the protocol

The statistician (DS), principal investigator (EAO), co-investiagotrs (KI), trial manager and research nurse are blind to treatment allocation.

Adherence to protocol can only be assessed by assessing wear time of the wearable technology. We will calculate the percentage of days the wearable technology was worn to measure adherence. A non-wear day is indicated by a blank output by the software while a figure of zero indicates no steps taken for the proportion of data recorded. If the wearable was not worn for 25% of the days during the observation period, then we will define it as non-adherence to treatment used for the CACE analyse on page 19.

## Loss to follow-up and other missing data

It is the aim of the trial to minimise withdrawal of participants within a treatment arm from treatment and follow-up. Withdrawal from the trial follow-up will be reported by intervention group. Summaries will be given of the reasons for withdrawal (grouped as appropriate). The distribution of times between randomisation and withdrawal from follow-up will be summarised in a histogram.

Where available, the reasons for missing baseline and follow-up data will be summarised overall and by randomised group at the visits and scale levels. Missing data in the database will be presented in compliance with CONSORT diagram.

### Missingness pattern of main outcome variable

The relationship between characteristics (demographic and clinical) and missing data will be summarised at 6 and 12 months follow-up (graphically) and the potential demographic and clinical factors that may be related to be missingness of HBA1c at 12 months will be examined.

## Adverse event reporting

Summaries adverse events (AE), adverse reactions (AR), serious adverse events (SAE) and serious adverse reactions (SAR), by intervention arm and time point (prior to 6 and 12 months) will be presented.

# Statistical Data Analyses Plan: Comparison of HBA1c between intervention groups (Primary outcome analysis)

## General comments

The main statistical analyses are targeted at estimating the difference in the mean outcomes between participants randomised to i) usual care and ii) usual care plus the DATES intervention at the 12 months post-treatment observation time. The primary outcome variable is HBA1c which is measured at baseline, 6- and 12-month follow-up.

The analyses of effectiveness outlined in this strategy will be pragmatic, based on intention-to-treat and will utilise all available follow-up data from all randomised participants. The trial statistician will remain blind whenever possible until the main analyses are complete. The significance level will be 5% (two-sided) for specified analyses and the guidelines of the CONSORT group for non-pharmacological clinical trials will be followed (Boutron, I., et al. 2008).

## Comments to Study design

The study design is moderately complex. Patients will be referred from two main recruiting centres: those who are registered at the DDI, which is a tertiary diabetes institute, and its affiliated primary care centres and other primary care centres in the rest of Kuwait that have electronic lists of diabetes patients that agree to participate. We assume that there are main differences between the i) DDI and ii) the other GP centres. Therefore, a fixed factor “Setting” with two levels needs to be included in the analyses model to account for potential difference between the two types of recruitment centres.

In addition, we expect differences between Kuwaiti national or Kuwaiti non-national and this factor “Nationality” also need to be considered as a fixed factor in the analysis model. Post-randomization measurements are taken at 6 month and 12 months follow-up and the repeated observations over time need to be accounted for in the analysis model.

## Overview of analyses of primary outcome:

1. Primary Intention to treat analysis
2. Sensitivity of results due to potential imbalance of confounders at baseline
3. Sensitivity of results due to missing data by
   1. including covariates predictive of missingness in analyses model
   2. assuming missing not at random
4. Sensitivity of results due to insulin uptake during the treatment period
5. Complier average causal effects (CACE)
6. Subgroup analyses

### Primary Intention to treat analysis

The main statistical analyses are targeted at estimating the difference in the mean HBA1c outcome i) between participants randomised to Treatment as usual and between participants randomised to DATES and Treatment as usual at 12 months follow-up observation time point.

An analysis of covariance (ANCOVA) approach is utilised for these analyses as the model accounts for the possible imbalance due to random sampling in baseline measurement of the outcome variable to control for pre-treatment differences. An analysis of covariance approach is preferred because of a usually increased statistical power to detect any treatment effects, since baseline and post-treatment measurements are assumed to be correlated. Furthermore, the ANCOVA approach is known to deal better with possible regression to the mean effects.

The main outcome values are assumed to arise from normal distributions (this will be checked - see Model assumptions checks - and if necessary appropriate transformations will be used). A linear mixed model using STATA’s *mixed* command will be used for estimation.

The outcome variables are HbA1c at 6 and 12 months after randomization. This allows us to include subjects with measurements with at least one observation assuming missingness at random.

In the linear mixed model “HbA1c” constitutes the dependent variable. “Treatment randomisation group”, “time (with two levels 6 and 12 months post-randomization)”, the interaction between “treatment group and time”, “Setting” (centre DDI v. non-DDI), “nationality”, the “baseline values of HbA1c”, and baseline covariate by time interactions are the fixed part of the model. “Time” will be entered as a categorical variable to avoid making a parametric assumption of the HbA1c levels over time.

To model the dependency of the repeated observations of the same subjects at 6 and 12 months we model the covariance between the residuals within the lowest level group” patients” to be correlated by using an unstructured covariance pattern model (Brown and Prescott 2006). If necessary, a different covariance structure will be estimated for each group.

For the final model, the group difference estimates and associated confidence intervals will be reported for 6 (for secondary analyses) and 12 months after randomization.

For the mixed effects model the following assumptions will be checked:

Normality: A histogram of level one standardised residuals against their normal scores will be produced to verify that the residuals have a normal distribution.

Outliers: Any obvious outliers or influential observations will be identified by the residuals and the analysis will be repeated excluding them to check the robustness of the conclusions of the primary analysis. Any differences when outliers are excluded will be reported.

Homogeneity of variances: A plot will be produced of the standardized residuals against the fitted values to examine the assumption of constant variability of the residuals across the range of fitted values. If necessary, we further relax the heteroskedascity assumption (error variance is the same between the two treatment groups) treatment specific error variances may be used.

If minor violations still exist, the results of the main analysis will be repeated and robust standard errors will be computed for inference.

### Sensitivity of results due to potential imbalance of confounders at baseline

The large sample size should ensure that all possible confounding variables are equally distributed between treatment arms. However, in a sensitivity analysis we extend the model of the primary analysis described in the previous section by including baseline variables thought to be important in determining outcome into the model. The analysis of this model will give intervention effect estimates adjusted for imbalances. We will report the changes in predicted outcome differences after controlling for all pre-specified baseline covariates simultaneously in addition to the main analysis model. Variables measured at baseline which we believe may affect outcome are:

1. Age
2. Gender
3. Nationality (already in main analysis model)
4. Education status
5. Marital status (Married, divorced, alone, widowed
6. Smoking status
7. Duration of diabetes
8. BMI
9. Insulin use/status
10. PHQ-9
11. PAID
12. HbA1c
13. IPAQ
14. HADS

### Sensitivity of results to missing data

There will be a certain proportion of patients for whom it is not possible to measure the main outcome variables at 6 or 12 months and the data are missing. We only expect very little or no missing data for at baseline.

The described mixed model will be fitted using maximum likelihood methods that are valid under the missing at random (MAR) assumption. However, this assumption relates to the variables that are included in the model, to allow for a variable predicting missingness this variable needs to be included as either one of the explanatory or dependent variables of the mixed model. This assumption will be investigated. Three types of sensitivity analysis for violations of the assumptions of MAR will be performed to assess the sensitivity of the results to missing outcome data.

**1. Sensitivity of results due to missing data by including covariates predictive of missingness in analyses model**

Should the investigation described in 2.5 indicate any demographic or clinical baseline variables that are predictors of outcome missingness, then such variables will be included as further covariates in the previous model and post-treatment group difference estimates and associated confidence intervals will be reported. We will assess the following variables:

- Age
- Gender
- Kuwait/no Kuwait citizen
- Education status
- Marital status (Married/Divorced/Alone/Widowed)
- Smoking status
- Duration of Diabetes
- BMI
- Self-reported physical activity (IPAQ)
- Clinical baseline values:
  - HbA1c
  - Hospital Anxiety and Depression Scale (HADS)
  - Patient Health Questionnaires 9 (PHQ9)
  - Problem Areas in Diabetes measure of diabetes specific quality of life (PAID)

**2. Sensitivity of results due to missing data by assuming missing not at random**

Analysis of data where the outcome is incomplete always requires untestable assumptions about the missing data commonly that they are missing at random. We will perform sensitivity analyses to explore the effect of departures from the assumption of missing at random made in the main analysis as recommended by White et al (2011) to explore the effect of departures from the assumption of Missing at random made in the main analysis. Specifically, we will assess how large an amount should be added to or subtracted from imputed outcomes without changing the clinical interpretation of the trial. We will report the “tipping point”, the value of the sensitivity parameter ∆ for which the main results are substantively affected (White et al 2017). The data and missingness are modelled jointly using a pattern-mixture model (modelling the differences between missing and observed data, using the user-written STATA package *rctmiss* (White 2017)

### Sensitivity of results due to insulin uptake during the treatment period

During the study people whose diabetes gets worse may be prescribed insulin which lowers their HbA1c level. A successful new treatment may prevent more people to get insulin prescribed in comparison to TAU. This could result in masking a positive treatment effect and may result in even overall lower HBA1c levels in the TAU group. We therefore perform a mediation analyses with uptake of insulin at 6 months as a mediator between treatment and main outcome at 12 months. This would allow us to separate the total observed treatment effect into the direct effect of treatment and the indirect effect via insulin uptake).

| **Figure 1**. Mediation model to estimate the direct effect of treatment on HBA1c at 12 months follow-up using a path analyses modelling approach using Mplus. Circles are error terms. | | |
| --- | --- | --- |
| Baseline | Six-month follow-up | 12-month follow-up |
| 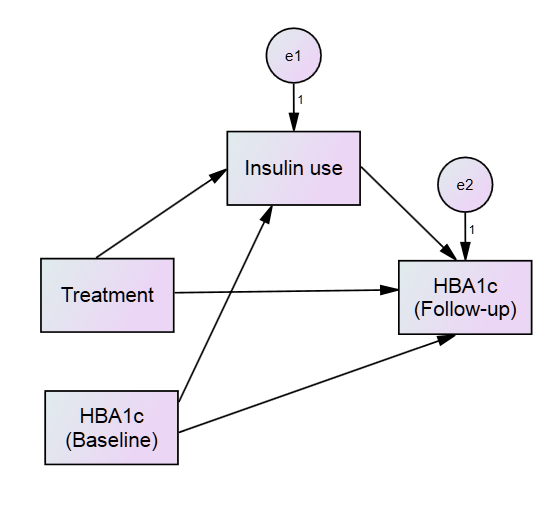 | | |

### Complier average causal effects (CACE)

In addition to the standard intention-to-treat analysis we will consider to estimate a measure of the treatment impact only for compliers as a secondary analyses. Specifically, we will employ instrumental variable (IV) methods to evaluate the causal effect of treatment in the subpopulations that are considered compliers to treatment. This complier average causal effect (CACE) is of scientific and policy interest, because it assesses the intervention effectiveness of the treatment when it is in fact taken (treatment *efficacy)*. The effect of not wearing the wearable wristband will result in less overall adherence to treatment and will be used as an indicator for compliance. . Compliance is defined as wearing the wearables for at least 25% of the days (see 2.4, page 12).

Noncompliance is a common problem in randomised clinical trials (RCT), which could cause biased results in determining the (non-ITT) effect of the treatment. Participants who comply with a particular treatment may be a biased sample of participants randomised to that treatment. Standard treatment effect analyses such as per-protocol (PP) analysis, which compares the average treatment effects for participants who comply with the assigned treatment, are therefore subject to bias. We therefore use an instrumental variable approach as suggested by Dunn et al (2005) to estimate the CACE at 12 months. In this approach randomisation indicator is used as an instrumental variable and treatment adherence as a binary treatment effect mediator between randomisation indicator and outcome. This approach provides a direct estimate of treatment efficacy which is protected from selection bias by the randomization. Analyses will be performed using STATA’s user written package ivreg2 (Baum et al 2007).

### Subgroup analyses

A treatment may be beneficial in some patients but may not provide benefits in others. An explorative subgroup analyses will be performed to identify potential modifiers of treatment response by including the following pre-specified variables in the model. A treatment arm by variable interaction will be assessed.

Potential effect modifiers:

- Depression
- Gender
- Insulin
- Duration of diabetes

In addition, we plan a sensitivity analyses to assess a moderating effect of setting on treatment outcome.

# Secondary outcome analyses

Treatment effects on secondary outcomes at 6 and 12 months will be assessed in a similar way as described in 3.2.1, using generalisations of the linear mixed model to allow for non-normal distributed data where necessary. The secondary outcomes are:

- Body mass index (BMI),
- Physical activity levels (number of steps and IPAQ)
- Fasting lipids
- Depressive symptoms (PHQ-9)
- Diabetes-specific quality of life (PAID)

# Considering incomplete measures of scale (questionnaires) data

This paragraph is not relevant for the primary outcome variable but only for some of the secondary outcome and baseline variables which are based on questionnaires or similar item-based measurement scales. The planned strategy for handling missing data at the item and scales will depend on the amount of missing data observed and the planned analyses for the outcomes.

Missing covariate item data will be imputed using prorating, that is by replacing the missing item score by the mean of the observed items if less than 20% of the items scores are missing. Items within each scale are indicators of a specific concept and as a result assumed to be closely and positively correlated and therefore regarded as a particularly applicable technique if less than 20 % are missing [Downey &King 1998, Roth et al 1999, Fox-Wasylyshyn and El-Masri 2005]. Simulation studies have shown that pro-rating (or case mean substitution) is a robust method when data are missing on less than 20% of items in both random and systematic patterns [Roth et al 1999].

Pro-rating is implemented across items within a scale, or subscale, for each assessment and participant. In the unlikely event that more than 20% missing items on at least some of the data collected multiple imputations will be implemented at the scale level. If this is carried forward it is essential that the item level data is not imputed by prorating at any point. Multiple imputation will also be considered if an item is missing in more than 5% of the patients who were administered the questionnaire.

To ensure the same strategy is followed across all scales reported in the principle paper(s), any guidance given by authors of validated questionnaires will supersede the methods outlined herein.

# Adverse Events

These analyses are based in the safety data and describe the safety outcomes. SAEs will be defined in accordance with the EU clinical trial Directives. Any adverse event deemed to be an SAE will initially be reported to the CI and then to the KCL (sponsor) within 48 hours. SAEs will be recorded by time and intervention group.

The following potential adverse events are monitored:

- Cardiovascular event
- Results in death;
- Is life-threatening;
- Required hospitalisation or prolongation of existing hospitalisation;
- Results in persistent or significant disability or incapacity

AEs will be tabulated by event type and clinical classification by time and randomised group. Each table will detail the number of participants that were still in the trial at the time points by randomisation group. If any adverse events are selected as being of particular interest they will be further summarised; outlining the severity (mild, moderate, severe) and if classified serious the expectedness (expected, unexpected) of the event. Any death will be reported as a serious adverse event.

Withdrawal from treatment will be regarded as potentially safety related outcomes and will be reported in terms of the reason, days from randomisation and person responsible for making the decision to withdraw.

# Compliance TO PROTOCOL

We will estimate the possible level of compliance as a potential source of bias but as this not be an accurate measure we will not summarise results in terms of compliance/ non-compliance. All unplanned unblindings of the researchers carrying out the quantitative assessments will be reported.

Chi-squared (or Fisher’s exact) tests will be used to describe any differences in withdrawals and SAEs between intervention groups.

# References

Baum, C.F., Schaffer, M.E., and Stillman, S. (2007) Enhanced routines for instrumental variables/GMM estimation and testing. The Stata Journal 7(4), 465-506.

Brown HK, Prescott RJ. (2006) Applied Mixed Models in Medicine, 2nd edition. John Wiley and Son.

Boutron, I., et al., (2008) Extending the CONSORT statement to randomized trials of nonpharmacologic treatment: Explanation and elaboration. Annals of Internal Medicine, 148(4): p. 295-309.

van Buuren, S. (2010) Multiple Imputation of Multilevel Data Handbook of Advanced Multilevel Analysis, ed. j.R. Hox, JK: Routledge Academic

Downey, R.G., & King, C.V. (1998). Missing data in Likert ratings: A comparison of replacement methods. Journal of General Psychology, 125, 175-191.

Dunn, G., Maracy, M. & Tomenson, B. (2005). Estimating treatment effects from randomized clinical trials with non-compliance and loss to follow-up: the role of instrumental variable methods. Statistical Methods in Medical Research 14, 369-395.

Fox-Wasylyshyn, S.M._and El-Masri, M.M.(2005) Focus on Research Methods Handling Missing Data in Self-Report Measures, Research in Nursing & Health, 2005, 28, 488-495:

Roth, P.L., Switzer, F.S., & Switzer, D.M. (1999) Missing data in multiple item scales: A Monte Carlo analysis of missing data, Organizational Research Methods 2 (3), 211-232

Royston, P. (2006) Multiple imputation of missing values: update. Stata Journal. 5(2): p. 188-201.

Schulz, K.F., Altman, D.G., Moher, D. for the CONSORT Group. CONSORT (2010) Statement: updated guidelines for reporting parallel group randomised trials. BMC Medicine , 8:18. (24 March 2010)

Shafer, J. (1997) Analysis of Incomplete Multivariate Data, 1997, New York: Chapman and Hall

White, I. R. & Carlin, J. B. (2010). Bias and efficiency of multiple imputation compared with complete-case analysis for missing covariate values. Statistics in Medicine 29, 2920–2931.

White I.R., Horton N., Carpenter J. & Pocock S.J. (2011). An intention-to-treat analysis strategy for randomised trials with missing outcome data. BMJ 342:d40.

White, I.A. (2017) "[RCTMISS: Stata module to analyse a randomised controlled trial (RCT) allowing for informatively missing outcome data](https://ideas.repec.org/c/boc/bocode/s458304.html)," [Statistical Software Components](https://ideas.repec.org/s/boc/bocode.html) S458304, Boston College Department of Economics.

White IR, Carpenter J, Horton NJ. A mean score method for sensitivity analysis to

departures from the missing at random assumption in randomised trials.

**arXiv:1705.00951v1**
